# Effects of anti-gonadotropin-releasing factor vaccination and administration age on the meat characteristics of pork loins from finishing gilts

**DOI:** 10.1016/j.vas.2025.100498

**Published:** 2025-08-19

**Authors:** Ditpon Kotatha, Narut Thanantong, Sukanya Phuengjayaem, Bing-Zheng Li, Alongkot Boonsoongnern, Autchara Kayan, Montri Pattarapanawan

**Affiliations:** a Division of Biochemical Technology, School of Bioresources and Technology, King Mongkut’s University of Technology Thonburi (Bangkhunthian Campus), Bangkok 10150, Thailand; b Department of Farm Resources and Production Medicine, Faculty of Veterinary Medicine, Kasetsart University (Kamphaeng Saen Campus), Nakhon Pathom 73140, Thailand; c Department of Microbiology, Faculty of Science, King Mongkut’s University of Technology Thonburi, Bangkok 10140, Thailand; d Institute of Grand Health, Guangxi Academy of Sciences, Nanning 530007, China; e Department of Animal Science, Faculty of Agriculture, Kasetsart University, Bangkok 10900, Thailand; f Department of Anatomy, Faculty of Veterinary Medicine, Kasetsart University, Bangkok 10900, Thailand

**Keywords:** Anti-gonadotropin-releasing factor, Immunocastration, Gilt, Pork, Meat quality

## Abstract

This pilot study evaluated the effects of anti-gonadotropin-releasing factor (anti-GnRF) vaccination and administration age on gilts (female pigs), with a specific focus on meat characteristics, including nutritional composition, meat quality, and the muscle histology of pork loins. A total of 66 Duroc × (Landrace × Large White) gilts were randomly assigned to three experimental groups: (1) intact gilts; (2) immunized gilts receiving a typical vaccination at 12 and 16 weeks of age; and (3) immunized gilts receiving early vaccination at 9 and 13 weeks of age, which is the earliest schedule recommended by the manufacturer. The pigs were slaughtered at the market age of 24 weeks, with meat samples collected from all of the groups for analysis, including meat samples from intact gilts (MIG), meat from gilts vaccinated at 12 and 16 weeks (MV12/16), and meat from gilts vaccinated at 9 and 13 weeks (MV9/13). Meat from the immunized gilts had significantly higher intramuscular fat (IMF) content than MIG (*P* < 0.05), accompanied by changes in the fatty acid profile—excluding polyunsaturated fatty acid—without adverse effects on meat quality or muscle histology. No significant differences were observed between the MV12/16 and MV9/13 samples. These findings suggest that anti-GnRF vaccination enhances the IMF content in pork without affecting meat quality or muscle structure. In addition, the administration age did not influence the chemical composition, meat quality, or muscle histology. An early vaccination schedule provides a practical approach to improve gilt production management, facilitating easier pig handling, efficient vaccination administration, and enhanced IMF deposition.

## Highlights

• The meat characteristics of anti-GnRF vaccinated gilts were evaluated in detail.
• Anti-GnRF vaccination increased the intramuscular fat content in gilts.
• Early vaccination had no adverse effects on meat characteristics.

## 1 Introduction

Immunocastration, which uses an anti-gonadotropin-releasing factor (anti-GnRF) vaccine, is an alternative form of animal welfare compliance to traditional surgical castration. This method has been used in more than 60 countries, including Australia, Mexico, Brazil, several European Union countries, Korea, and Thailand (Ayalew, 2019). The vaccine stimulates the production of antibodies that bind to and neutralize endogenous gonadotropin-releasing hormone (GnRH), inactivating the gonadal function that secretes sex hormones (estrogen and progesterone) (Allison et al., 2021). This occurs by suppressing the follicle-stimulating hormone (FSH) and luteinizing hormone (LH) from the pituitary gland (Hernández-García et al., 2013).

Immunocastration is commonly used in commercial pig production, particularly for boars (male pigs). The main aim is to reduce boar taint, a major factor causing consumer rejection. This undesirable smell is mainly caused by the accumulation of androstenone and skatole during puberty (Moore, 2024). Androstenone is a testicular steroid that produces a urine or sweat-like odor. Skatole is a microbial metabolite of tryptophan absorbed from the colon (Fuchs et al., 2009). Immunocastration also has secondary benefits, including increased feed intake, growth performance, and fat deposition, and a reduction in aggressive behavior (Van den Broeke et al., 2022). Numerous studies have investigated the effects of immunocastration on production outcomes in boars, with findings summarized in a recent meta-analysis (Batorek et al., 2012; Nautrup et al., 2018; Pauly et al., 2012). The effects of immunocastration on meat quality in male pigs have also been extensively studied, with a meta-analysis conducted on this subject (Batorek et al., 2012). While immunocastration is mainly used for boars, it has also been used for gilts (female pigs) to suppress ovarian function and inhibit the estrous cycle, particularly in pigs raised for heavier slaughter weights (Small et al., 2023), and offers similar benefits to those observed in boars (Bohrer et al., 2014; Hinson et al., 2012). In Spain, immunocastration has been used for Iberian gilts to prevent pregnancies resulting from cohabitation with wild boars in extensive farming systems (Dalmau et al., 2015). In Thailand, the vaccine’s use in commercial crossbred Duroc × (Landrace × Large White) gilts has recently increased in intensive production systems. However, few peer-reviewed studies have investigated the effects of immunocastration on meat quality in gilts.

**Fig. 1:**
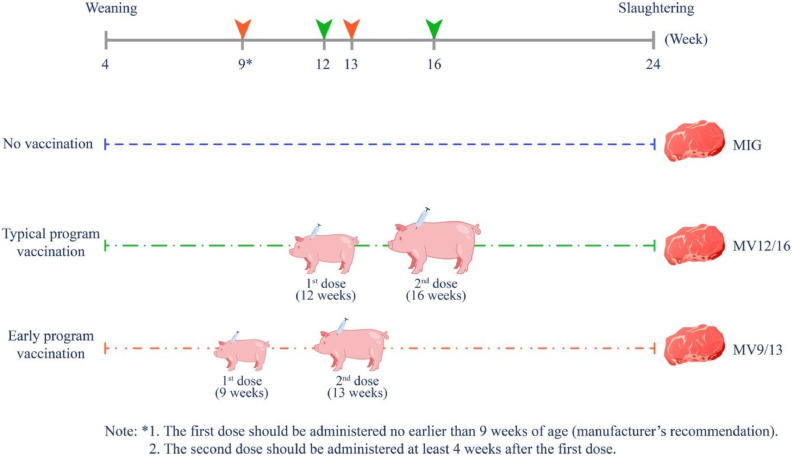
Schematic overview of the vaccination schedules and corresponding meat samples. Abbreviations: MIG = meat from intact gilts; MV12/16 = meat from immunized gilts vaccinated at 12 and 16 weeks of age; MV9/13 = meat from immunized gilts vaccinated at 9 and 13 weeks of age.

The manufacturer’s instructions (ZoetisUS, 2020) recommend two doses of the anti-GnRF vaccine for gilts, with the first dose administered no earlier than 9 weeks of age, and the second dose given at least 4 weeks after the first. This regimen has been shown to effectively suppress estrus between 3 and 10 weeks after the second dose. This program is designed to ensure that antibody levels are sufficient to neutralize endogenous GnRH prior to the onset of puberty, which generally occurs between 150 and 220 days of age (Soede et al., 2011). Furthermore, Dalmau et al. (2015) reported that the vaccine maintained a low circulating progesterone level in Iberian gilts for up to 12 weeks after administration of the second dose. In Thailand, anti-GnRF vaccination typically takes place at 12 (first dose) and 16 (second dose) weeks of age in gilts, similar to studies in Vietnam (Lai et al., 2024), Spain (Daza et al., 2016), and the United States (Bohrer et al., 2014). However, administering the vaccine to heavier pigs presents significant challenges due to the greater physical strength and behavioral reactivity, requiring more staff, specialized equipment such as snares. These procedures can induce substantial stress, which in turn may suppress appetite (Figueroa et al., 2015), compromise immune function (Magnusson et al., 1998), and potentially compromising animal welfare. Applying an earlier vaccination schedule, at 9 and 13 weeks of age, which is the earliest recommended by the manufacturer, may provide practical advantages. Younger and lighter pigs can be restrained more easily with basic tools like small pens or handheld devices. A single trained handler can often administer the vaccine effectively, with minimal risk to both the animal and the operator. The typical and early vaccination schedules are illustrated in Fig. 1.

This pilot study investigated the effects of anti-GnRF vaccination and vaccination timing on finishing gilts, focusing on the nutritional composition, meat quality traits, and muscle histology of pork loin. Vaccinations were either administered following the typical schedule at 12 and 16 weeks of age or an earlier schedule of 9 and 13 weeks. The hypothesis was that the vaccine administration might influence on the pork loin characteristic, while vaccination timing has no significant effect. The findings provide valuable insights, providing a potential alternative strategy for gilt finishing management to improve the practicality in pork production systems.

## 2 Material and methods

### 2.1 Ethics approval

All experiments were strictly conducted according to the guidelines for animal care and use under the Ethical Review Board of the Office of National Research Council of Thailand (NRCT) and approved by the Institutional Animal Care and Use Committee of Kasetsart University with letter of approval ID. ACKU67-VET-059

### 2.2 Animal husbandry and meat sample collection

A total of 66 healthy weaned Duroc × (Landrace × Large White) gilts with live weights of between 6 and 7 kg were obtained from Charoen Pokphand Foods Public Company Limited (Thailand). The pigs were fed ad libitum with a basal diet formulated to meet the nutritional standards of the National Research Council (2012). The basal diet comprised three phases—starter, grower, and finisher—and was based on a commercial feed obtained from Cargill Co., Ltd. (Thailand). The water consumed was supplied ad libitum through nipple drinkers.

The study was conducted using a completely randomized design (CRD), with the 66 gilts allocated to three groups of 22 animals each. Each group was housed in two pens, with 11 pigs per pen, and each pen measured 4 × 5 m. The three groups comprised an intact group and two groups of immunized gilts with different vaccination timings: typical vaccination (12 and 16 weeks) and early vaccination (9 and 13 weeks). Immunocastration comprised the administration of a 2 mL dose of anti-GnRF vaccine (Improvac®, Zoetis Inc.) via intramuscular injection on the lateral neck surface. The control pigs did not receive a placebo, following the approach of Van den Broeke et al. (2016), because the study aimed to evaluate the comprehensive effects of the anti-GnRF vaccination process, including the injection procedure, the administered conjugate, adjuvants, active ingredient, and both specific and nonspecific immune responses induced by the vaccine. The use of a non-treated control group allowed for the assessment of the overall biological impact of immunocastration under practical field conditions, without isolating individual components of the vaccine formulation.

The gilts were slaughtered at 24 weeks of age following a 12-h feed withdrawal. The distance from the farm to the slaughterhouse was 30 km. The gilts from the same pen were kept together in the same lairage room before slaughter. Stunning was performed via head-only electrocution, followed by exsanguination, scalding, dehairing, and evisceration following standard commercial protocols. Meat samples were then collected from the *longissimus thoracis et lumborum* (LTL) on the left side of each carcass, extending 15 cm around the thirteenth rib. Five gilts from each experimental group with live body weights closest to the pen average were selected, producing 10 samples per group (5 animals per pen × 2 pens). The meat samples were cut to a 2.5 cm thickness and trimmed of visible fat. Fresh samples were assessed for pH at 45 min post-mortem (pH_45_) and muscle histology then stored at 4 °C for analysis of ultimate pH (pH_u_), color, and water holding capacity (WHC). Additional samples were vacuum-sealed and stored at −20 °C until further analysis. Meat from the intact gilts, meat from immunized gilts vaccinated at 12 and 16 weeks, and meat from immunized gilts vaccinated at 9 and 13 weeks were designated MIG, MV12/16, and MV9/13, respectively. A schematic overview of the vaccination schedule and meat samples is shown in Fig. 1.

### 2.3 Nutritional composition

#### 2.3.1 Chemical composition of meat

The meat samples with visible fat removed, stored at −20 °C, were thawed at 4 °C for 24 h and then ground using a food blender (E3HB1–880 K, Electrolux, Thailand). The moisture content was then determined following the Association of Official Analytical Chemists (AOAC International, 2003) method 930.15. The remaining meat sample portion was dried at 50 °C for 24 h for protein, ash, and crude fiber content analysis. The protein content was determined using a nitrogen-to-protein conversion factor of 6.25 following the AOAC method 984.13. The ash and crude fiber contents were determined following the AOAC methods 942.05 and 962.09, respectively. The intramuscular fat (IMF) content was calculated based on the total lipid content, which was determined using the Soxhlet method following the AOAC method 2003.05.

#### 2.3.2 Fatty acid profile of intramuscular fat

The IMF for fatty acid profile analysis was extracted from the ground meat samples using a mixture of chloroform and methanol following Bligh & Dyer (1959), and the fatty acid methyl esters (FAMEs) were prepared following Pojjanapornpun et al. (2018). The FAMEs were analyzed using gas chromatography (GC-2010, Shimadzu, Japan) combined with a flame ionization detector, with a BPX70 capillary column (70 % bis-cyanopropyl-polysilphenylene-siloxane, 30 m length, 0.25 mm internal diameter, and 0.25 µm film thickness). Nonadecanoic acid was used as an internal standard. The oven temperature was set at 160 °C for 2 min and was increased to 210 °C at a rate of 2 °C/min for 20 min. Helium was used as the carrier gas at a flow rate of 1 mL/min. The temperature of the injector was set at 250 °C, with an injection volume of 1 µL with a split ratio of 100:1. Fatty acid content was reported as proportions and as absolute amounts (mg/100 g of LTL).

### 2.4 Meat quality

#### 2.4.1 pH of meat

The pH_45_ of the meat samples was measured using a meat pH meter (HI981036, Hanna, United States) at room temperature. The samples were then stored at 4 °C for 24 h. Following refrigeration, the samples were equilibrated to room temperature for 30 min before measuring the pHᵤ. Before measurement, the pH meter was calibrated using standard buffers at pH 4.01 and 7.00 (Thermo Fisher Scientific, United States). The mean of five random measurements taken at different points in a sample was used to represent the sample.

#### 2.4.2 Meat color

The surface color of the meat samples was determined using a colorimeter (MiniScanEZ 4500, HunterLAB, United States) after 24 h of cooling at 4 °C. The meat samples were allowed to bloom at room temperature for 30 min before measurement. The colorimeter was calibrated using white and black standard plates and configured to the CIE LAB color system. The colorimeter was set to a D65 illuminant, a 10° standard observer angle, and a 2.5 cm aperture. The color parameters were reported using the CIELAB system: L* (lightness), a* (redness), b* (yellowness), C* (chroma), and *h* (hue angle). The mean of five random measurements taken at different points in a sample was used to represent the sample.

#### 2.4.3 Water holding capacity

The WHC—including drip loss, thawing loss, and cooking loss—was measured. Drip loss was determined using the suspension method by Nollet & Toldrá (2008) with slight modifications. The meat samples were cut into pieces measuring 5 × 5 × 2 cm and weighed. Each sample was wrapped in a gauze and suspended inside a polyethylene bag, ensuring that the gauze and the bag were not in contact. The samples were then stored at 4 °C for 48 h. Subsequently, the samples were removed, any surface moisture was absorbed using absorbent paper, and the final weights were measured. Drip loss was calculated using Eq. (1):

(1) $$\text{Drip}\;\text{loss}\;(\%)\;=\;\frac{\left(W1\;-\;W2\right)}{W1}\;\times \;100$$

where W1 is the weight of the sample before being suspending at 4 °C, and W2 is the weight of the sample after being suspending at 4 °C.

Following the drip loss assessment, the thawing loss was determined as described by Tippala et al. (2021) with slight modifications. The samples were sealed in vacuum bags and frozen at −20 °C for 48 h. The frozen samples were then thawed at 4 °C for 24 h. Following thawing, the samples were removed, any surface moisture was absorbed using absorbent paper, and the final weight was recorded. The thawing loss was calculated using Eq. (2):

(2) $$\text{Thawing}\;\text{loss}\;(\%)\;=\;\frac{\left(W2\;-\;W3\right)}{W2}\;\times \;100$$

where W2 is the weight of the sample before freezing at −20 °C, and W3 is the weight of the sample after thawing at 4 °C.

To assess cooking loss, the thawing loss samples were examined following Watanabe et al. (2018) with a slight modification. The samples were resealed in thin-walled bags and heated in a temperature-controlled water bath until their core temperature reached 75 °C. The samples were then cooled to room temperature. Following cooling, the samples were removed from the bags, and any surface moisture was absorbed using absorbent paper. The final weight of each sample was then recorded. Cooking loss was calculated using Eq. (3):

(3) $$\text{Cooking}\;\text{loss}\;(\%)\;=\;\frac{\left(W3\;-\;W4\right)}{W3}\;\times \;100$$

where W3 is the weight of the sample before cooking, and W4 is the weight of the sample after cooking.

#### 2.4.4 Meat tenderness

Meat tenderness was assessed using the Warner–Bratzler shear force method, adapted from U-chupaj et al. (2017). Meat samples stored at −20 °C were thawed at 4 °C for 24 h and then placed in vacuum-sealed bags, ensuring an airtight seal. The samples were then cooked in a temperature-controlled water bath until their core temperature reached 75 °C. The samples were subsequently cooled to room temperature for analysis. The samples were cut into 1 × 1 × 2 cm pieces and analyzed using a texture analyzer (TA-XT plus, Stable Micro Systems, United Kingdom) equipped with a 500 N load cell and a 3 mm thick Warner–Bratzler blade. The samples were sheared perpendicular to the fiber orientation at a crosshead speed of 200 mm/min and a working distance of 25–30 mm. Shear force was measured using 10 pieces per meat sample, following the guidelines of the American Meat Science Association (2015). The highest and lowest values were excluded when calculating the average shear force (Kowalski et al., 2021). A total of 80 pieces were analyzed per experimental group. The peak shear force was recorded in newtons (N).

#### 2.4.5 Lipid oxidation (thiobarbituric acid reactive substances)

Lipid oxidation was assessed using the thiobarbituric acid reactive substances (TBARS) method following the protocol outlined by Rossi et al. (2013) with slight modifications. The meat samples stored at −20 °C were first thawed at 4 °C for 24 h and then ground using a food blender. Approximately 5 g of ground meat was then transferred to a 50 mL centrifuge tube and stored in a refrigerator at 4 °C for 7 days. Next, 15 mL of 10 % trichloroacetic acid solution was added to the tube, and the mixture was homogenized using a homogenizer (OV5, VELP Scientifica, Italy) and incubated for 1 h at room temperature in a shaking incubator (WNB22, Memmert, Germany). Following this, the homogenate was centrifuged at 5 000 × *g* at 4 °C for 10 min, and the supernatant was filtered using Whatman No. 1 filter paper. For the TBARS measurement, 5 mL of the supernatant was mixed with 5 mL of 0.02 M thiobarbituric acid solution. The mixture was then heated in boiling water for 35 min with frequent vortex mixing before being immediately chilled in an ice bath. The final solution’s absorbance was measured at 532 nm using a spectrophotometer (Genesys 20, Thermo Fisher Scientific, United States). A standard curve was constructed to quantify the TBARS value based on 1,1,3,3-Tetramethoxypropane (MDA), which was recorded as milligrams of MDA equivalent per kilogram of sample (mg MDA/kg).

### 2.5 Muscle histology

The meat samples were cut into 1 cm^3^ cubes and fixed with 4 % paraformaldehyde. Next, 4 µm-thick sections of the paraffin-embedded samples were prepared and stained with hematoxylin and eosin (Pattarapanawan et al., 2020). The histological structures of the sample cross-sections were examined using a light microscope (Axiolab 5, Zeiss, Germany) equipped with a 10 × objective lens and a 10 × eyepiece, and ZEN 3.4 photographic software (Zeiss, Germany). Five images were captured from different cross-sections of each sample. Image analysis was performed using ImageJ software (Schneider et al., 2012), and the histological parameters were evaluated following Koomkrong et al. (2015). The mean number of muscle fibers per unit area was determined by counting the total number of fibers in the 500 000 µm^2^ area of each photograph. The cross-sectional areas (µm^2^) and diameters (µm) of approximately 300 muscle fibers were assessed in each photograph. The mean thicknesses (µm) of the perimysium and endomysium were calculated from 10 and 40 measurements, respectively, from each photograph.

### 2.6 Statistical analysis

Statistical analyses for all parameters were performed using a linear mixed model. The three experimental groups were treated as fixed effects, while the animal pen was considered a random effect. Statistical significance was set at *P* < 0.05. Post hoc multiple comparisons of means were conducted using the Bonferroni test (*P* < 0.05). Each experimental group comprised 10 samples (*n* = 10). The number of replications varied depending on the analysis type as follows: fatty acid profile and WHC (two replicates); chemical composition and lipid oxidation (three replicates); pH and color measurements (five replicates); and Warner–Bratzler shear force (10 replicates). For the muscle histology analysis, five regions were selected from the different cross-sections of each muscle sample. All statistical analyses were conducted using SPSS Statistics software (version 18, IBM Corp., United States).

## 3 Results

### 3.1 Nutritional composition

The chemical compositions of the MIG, MV12/16, and MV9/13 samples are presented in Table 1. Moisture, protein, and ash levels did not differ significantly among the groups. Crude fiber was not detected in any of the groups. The IMF content was 1.81 % in the MIG samples compared with 2.49 % and 2.45 % in the MV12/16 and MV9/13 samples, respectively, meaning that the IMF content was significantly higher in the meat from the immunized gilts than from the MIG group (*P* < 0.05). Furthermore, altering the vaccination schedule from the typical program (12 and 16 weeks) to early vaccination (9 and 13 weeks) had no significant effect on IMF content, with no difference observed between the MV12/16 and MV9/13 samples.

**Table 1:** Chemical compositions of MIG, MV12/16, and MV9/13.

Chemical composition	% in fresh matter				SEM	*P*-value
	MIG	MV12/16		MV9/13		
Moisture content	72.9	72.1	72.6		0.31	0.184
IMF	1.81^b^	2.49^a^	2.45^a^		0.026	0.019
Protein	24.0	24.1	24.1		0.21	0.881
Ash	1.17	1.14	1.22		0.019	0.523

Note: Crude fiber was not detected in any of the groups.

Abbreviations: MIG = meat from intact gilts; MV12/16 = meat from immunized gilts vaccinated at 12 and 16 weeks of age; MV9/13 = meat from immunized gilts vaccinated at 9 and 13 weeks of age; IMF = intramuscular fat.

The notation “a, b, c, …” indicates significant differences between the groups at *P* < 0.05, while the absence of notation indicates no significant differences.

The fatty acid profiles of the IMF from the three experimental groups are shown in Table 2. No significant differences were observed between these groups regarding the proportions ( % of total) of saturated fatty acids (SFA), monounsaturated fatty acids (MUFA), and polyunsaturated fatty acids (PUFA). However, based on the absolute fatty acid content per 100 g of LTL, the MV12/16 samples had significantly higher SFA and MUFA levels compared with the MIG samples (*P* < 0.05). In contrast, the MV9/13 samples showed comparable values to those of the MV12/16 and MIG groups. The PUFA content was comparable among the three experimental groups.

**Table 2:** Lipid profile of IMF of MIG, MV12/16, and MV9/13.

	Proportions of fatty acid (%)			SEM	*P*-value	Absolute amounts of fatty acid (mg/100 g of LTL)			SEM	*P*-value
	MIG	MV12/16	MV9/13			MIG	MV12/16	MV9/13		
C14:0	1.14	1.37	1.17	0.045	0.130	20.59^b^	34.16^a^	27.42^ab^	2.242	0.001
C16:0	23.2	25.8	23.3	0.81	0.338	419.3^b^	642.7^a^	544.4^ab^	43.73	0.006
C16:1n7	1.86^b^	2.56^a^	1.91^b^	0.055	0.014	33.87^b^	63.70^a^	44.57^b^	3.917	<0.001
C18:0	12.4	12.7	12.2	0.44	0.868	223.4^b^	316.8^a^	285.7^ab^	22.44	0.025
C18:1n9	37.9	40.0	37.3	1.39	0.640	683.7^b^	996.7^a^	870.3^ab^	69.37	0.016
C18:1n7	2.68	3.05	2.71	0.135	0.428	48.50^b^	76.05^a^	63.23^ab^	5.115	0.004
C18:2n6	10.6	8.8	11.0	0.50	0.215	192.4	220.0	256.4	18.18	0.065
C18:3n3	0.45	0.38	0.49	0.059	0.681	8.12^b^	9.47^ab^	11.43^a^	0.788	0.024
C20:0	0.31	0.18	0.17	0.057	0.487	5.599^a^	4.488^ab^	3.967^b^	0.403	0.029
C20:1	0.76	0.66	0.66	0.075	0.765	13.73	16.46	15.40	1.244	0.322
C20:2n6	0.48	0.36	0.52	0.057	0.446	8.58^b^	8.85^b^	12.02^a^	0.804	0.010
C20:3n6	0.20	0.12	0.21	0.015	0.106	3.612^b^	2.992^b^	4.900^a^	0.3187	0.001
C20:4n6	1.43^a^	1.00^b^	1.43^a^	0.048	0.024	25.83^ab^	24.93^b^	33.48^a^	2.308	0.027
SFA^1^	37.0	40.0	36.9	1.326	0.499	668.9^b^	998.1^a^	861.5^ab^	68.78	0.010
MUFA^2^	43.2	46.2	42.6	1.643	0.557	779.8^b^	1152.9^a^	993.5^ab^	79.62	0.012
PUFA^3^	13.2	10.7	13.6	0.672	0.204	238.5	266.3	318.3	22.39	0.057

Abbreviations: MIG = meat from intact gilts; MV12/16 = meat from immunized gilts vaccinated at 12 and 16 weeks of age; MV9/13 = meat from immunized gilts vaccinated at 9 and 13 weeks of age.

1 SFA = saturated fatty acid: C14:0 + C16:0 + C18:0 + C20:0.

2 MUFA = monounsaturated fatty acid: C16:1n7 + C18:1n9 + C18:1n7 + C20:1.

3 PUFA = polyunsaturated fatty acid: C18:2n6 + C18:3n3 + C20:2n6 + C20:3n6 + C20:4n6 The notation “a, b, c, …” indicates significant differences between the groups at *P* < 0.05, while the absence of notation indicates no significant differences.

### 3.2 Meat quality

The meat quality parameters—including pH, color, WHC, Warner–Bratzler shear force, and lipid oxidation—of the three experimental groups are shown in Table 3. Overall, no significant differences were observed between the three groups across all of the evaluated parameters. The pH_45_ and pH_u_ values did not differ significantly among the groups. The pH_45_ values were 6.33, 6.22, and 6.24, and the pHᵤ values were 5.71, 5.67, and 5.66 for the MIG, MV12/16, and MV9/13 samples, respectively. Similarly, no significant differences were observed in the color values, including L*, a*, b*, C*, and *h*. The WHC also showed comparable values among the three groups. The drip loss values were 4.1 %, 3.9 %, and 4.4 %; the thawing loss values were 11.2 %, 12.7 %, and 12.0 %; and the cooking loss values were 16.0 %, 17.2 %, and 18.0 % for the MIG, MV12/16, and MV9/13 samples, respectively. The Warner–Bratzler shear force, used to evaluate meat tenderness, produced values of 43.98, 43.66, and 52.73 N for the MIG, MV12/16, and MV9/13 groups, respectively, with no statistically significant differences. Lipid oxidation measured by TBARS also showed no statistically significant differences between the treatments—the TBARS values were 0.137, 0.131, and 0.127 mg MDA/kg for the MIG, MV12/16, and MV9/13 samples, respectively.

**Table 3:** Meat quality of MIG, MV12/16, and MV9/13.

	Parameter	MIG	MV12/16	MV9/13	SEM	*P*-value
pH						
	pH_45_	6.33	6.22	6.24	0.083	0.722
	pH_u_	5.72	5.67	5.66	0.038	0.554
Color						
	L*	53.6	54.6	56.1	0.93	0.182
	a*	7.99	8.60	7.16	0.442	0.403
	b*	14.2	15.0	14.5	0.35	0.246
	C*	16.4	17.4	16.3	0.43	0.398
	h	60.9	60.4	63.7	1.55	0.286
WHC						
	Drip loss (%)	4.08	3.89	4.58	0.516	0.623
	Thawing loss (%)	11.2	12.7	12.0	0.93	0.513
	Cooking loss (%)	16.0	17.2	18.0	1.15	0.698
Warner-Bratzler shear froce						
	Shear force (N)	44.0	43.7	52.7	3.25	0.100
Lipid oxidation						
	TBARS (mg/kg)	0.137	0.131	0.127	0.0033	0.677

Abbreviations: MIG = meat from intact gilts; MV12/16 = meat from immunized gilts vaccinated at 12 and 16 weeks of age; MV9/13 = meat from immunized gilts vaccinated at 9 and 13 weeks of age; pH_45_ = pH of meat at 45 min after slaughtering; pH_u_ = pH of meat after 24 h after slaughtering; L* = lightness; a* = redness; b* = yellowness; C* = chrome; *h* = hue; TBARS = thiobarbituric acid reactive substances.

The notation “a, b, c, …” indicates significant differences between the groups at *P* < 0.05, while the absence of notation indicates no significant differences.

### 3.3 Muscle histology

**Fig. 2:**
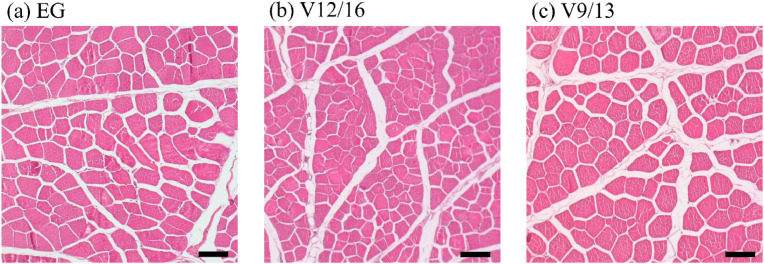
Histological cross-sections of the LTL of (a) MIG, (b) MV12/16, and (c) MV9/13. Scale bar = 100 µm. Abbreviations: MIG = meat from intact gilts; MV12/16 = meat from immunized gilts vaccinated at 12 and 16 weeks of age; MV9/13 = meat from immunized gilts vaccinated at 9 and 13 weeks of age.

The muscle histology of the LTL for the three experimental groups is shown in Fig. 2. The muscle fibers had typical angular outlines with stippled cytoplasm, and numerous basophilic, oval nuclei were located peripherally within the fibers. The perimysium surrounding the muscle fascicles contained vascular and adipose tissues, while the endomysium enclosed individual muscle fibers. The corresponding quantitative histological parameters are summarized in Table 4. The groups exhibited comparable values across all measured parameters, with no statistically significant differences detected. The total number of muscle fibers, cross-sectional diameters, and cross-sectional areas did not differ significantly between the treatments. Similarly, the perimysium and endomysium thicknesses showed no significant variations between the three groups.

**Table 4:** Muscle histological structure of MIG, MV12/16, and MV9/13.

Parameters	MIG	MV12/16	MV9/13	SEM	*P-*value
Total number of fibers	135.5	143.9	137.1	10.64	0.856
Cross-section diameter (µm)	38.82	50.50	40.87	7.129	0.468
Cross-section area (µm^2^)	2424	2080	2327	296.1	0.706
Perimysium thickness (µm)	41.10	51.13	46.67	4.880	0.352
Endomysium thickness (µm)	13.20	12.51	11.57	1.623	0.789

Abbreviations: MIG = meat from intact gilts; MV12/16 = meat from immunized gilts vaccinated at 12 and 16 weeks of age; MV9/13 = meat from immunized gilts vaccinated at 9 and 13 weeks of age.

The notation “a, b, c, …” indicates significant differences between the groups at *P* < 0.05, while the absence of notation indicates no significant differences.

## 4 Discussion

### 4.1 Nutritional composition

#### 4.1.1 Chemical composition of meat

The chemical compositions of the samples from the three experimental groups (Table 1) indicate that anti-GnRF vaccination significantly increased the IMF content of the meat, aligning with previous studies by Daza et al. (2014) and Van den Broeke et al. (2016). The anti-GnRF vaccine reduced the secretion of gonadotropins (FSH and LH) (Hernández-García et al., 2013), thereby suppressing fat deposition and IMF accumulation through the downregulation of follicle-stimulating hormone receptor (FSHR) and the peroxisome proliferator-activated receptor gamma (PPARγ) (Cui et al., 2012, 2016). In addition to gonadotropin suppression, the vaccine also decreases sex hormone (estrogen and progesterone) production (Allison et al., 2021). Interestingly, the absence of sex hormones influenced fat deposition by increasing increased feed intake (Van den Broeke et al., 2016), reducing the metabolic efficiency, which means that the body becomes less effective at converting nutrients into useable energy, resulting in deposition of adipose tissue (Ambikairajah et al., 2019; Comitato et al., 2015), and suppressing catecholamine signal transduction in fat cells (Arner, 2005). Therefore, the current findings may be attributed to the greater impact of the absence of sex hormones on fat accumulation than the direct effects of gonadotropin depletion. However, the effect of anti-GnRF vaccination on IMF content remains unclear, with several studies reporting no significant changes in the IMF in meat from immunized gilts (Bohrer et al., 2014; Daza et al., 2016; Gamero-Negrón et al., 2015; Martinez-Macipe et al., 2016; Pérez-Ciria et al., 2021). These inconsistencies may arise from various factors, such as differences in the relative physiological effects of sex hormone suppression versus gonadotropin inhibition, breed differences, or variations in slaughter age.

A higher IMF content enhances the characteristics of high-quality meat products, making it a positive and valuable attribute for the meat industry (Gamero-Negrón et al., 2015; Gandemer, 2002). In addition, elevated fat levels have a positive correlation with the sensory attributes of meat and meat products, including taste, flavor, tenderness, and juiciness (Essen-Gustavsson et al., 1994; Ruiz-Carrascal et al., 2000).

The MV12/16 and MV9/13 samples were retrieved from immunized gilts with different post-vaccination periods. The MV12/16 samples were taken from immunized gilts with an 8-week post-vaccination period, as they had received the second vaccine dose at 16 weeks and were slaughtered at 24 weeks. In contrast, the MV9/13 samples were taken from immunized gilts with an 11-week post-vaccination period, as they had received the second vaccine dose at 13 weeks and were slaughtered at 24 weeks (Fig. 1). Despite this difference in post-vaccination periods, no significant differences in IMF content was observed between the two groups. This may be attributed to the similar active serum gonadotropin levels in these groups due to the serum anti-GnRF, which was maintained for up to 12 weeks following vaccination (Dalmau et al., 2015). In addition to nutritional aspects, fatty acid composition has a crucial role in the nutritional quality of meat and adipose tissue. It has been extensively studied in meat science, reflecting its significance for human health. For these reasons, the composition of fatty acids in meat has become an important issue for consumers, nutritionists, and food technologists (Kasprzyk et al., 2015; Nuernberg et al., 2015).

#### 4.1.2 Fatty acid profile of intramuscular fat

Fatty acid synthesis is influenced through *de novo* lipogenesis pathway, primarily involving the conversion of acetyl-CoA into palmitic acid (C16:0) through a series of enzymatic reactions. Subsequently, palmitic acid can undergo further modifications, including elongation of the carbon chain by elongase enzymes and introduction of double bonds by desaturase enzymes, resulting in the formation of long-chain and unsaturated fatty acids, respectively (Al Rharad et al., 2025; Boukrouh et al., 2024; Dinh et al., 2021). The present results demonstrated that no differences were observed in the SFA, MUFA, and PUFA percentages in the IMF from three experimental group (Table 2) suggesting that anti-GnRF vaccination does not significantly influence the relative proportions of IMF fatty acids. This result aligns with Gamero-Negrón et al. (2015), who also reported no significant changes in IMF fatty acid proportions following anti-GnRF vaccination. However, when evaluating the absolute fatty acid content per 100 g of LTL, the MV12/16 samples had significantly higher amounts of SFA and MUFA compared with the MIG samples. This suggests that the typical vaccination schedule (at 12 and 16 weeks) may enhance fatty acid deposition, which is consistent with its observed effect on IMF content. In contrast, early vaccination (at 9 and 13 weeks) in the MV9/13 group did not significantly alter the amounts of SFA and MUFA compared with either the MIG or MV12/16 groups, indicating a limited impact on some fatty acid accumulation. Notably, the PUFA content remained unchanged in the three groups. The lack of significant changes in PUFA composition due to anti-GnRF vaccination is advantageous, as PUFA have a critical role in various aspects. For example, the PUFA proportion influences lipid oxidation, which affects shelf life, rancidity, and color deterioration (Wood et al., 2004). In addition, PUFA reduces serum low-density lipoprotein, which is a key factor associated with an increased risk of cardiovascular disease (Caldara et al., 2018).

### 4.2 Meat quality

Meat quality includes intrinsic properties that are crucial to its suitability for further processing, storage, and retail display. In this study, key attributes—including pH, color, WHC, texture, and oxidative stability—were evaluated.

#### 4.2.1 pH of meat

The pH_45_ and pHᵤ values of the meat samples did not differ significantly between the three experimental groups. This observation aligns with previous studies, which also reported similar pH₄₅ and pHᵤ values between meat from intact and immunized gilts (Martinez-Macipe et al., 2016; Van den Broeke et al., 2016; Xue et al., 2019). Following slaughter, meat pH gradually declines due to the accumulation of lactic acid produced through anaerobic metabolism (Pearce et al., 2011). The typical pH_u_ is approximately 5.5. This reduction in pH has a critical role in determining the color, texture, and WHC of the meat. Rapid decreases in pH_45_ to 5.4–5.5, together with slightly lower than normal pH_u_, produce undesirable characteristics, including pale color, soft texture, and exudative properties, which are typical of pale, soft, and exudative (PSE) meat. In contrast, meat with a pH_u_ greater than 6.0–6.2 has a dark, purple-red to almost black color and a dry surface, which are indicative of dark, firm, and dry (DFD) meat (Arias, 2012). In this study, the pH_45_ and pH_u_ values of the samples from the three experimental groups were close to those of normal meat quality. This suggests that the administration of the anti-GnRF vaccine and adjustments in the vaccination timing did not significantly affect the pH dynamics in the muscle tissue.

#### 4.2.2 Meat color

Color is a key characteristic of fresh meat and significantly influences consumer choice (Al Rharad et al., 2025; Boukrouh et al., 2024; Ngapo et al., 2007). Meat color is affected by various factors, including myoglobin (Mb) concentration, Mb chemical state (deoxyMb, oxyMb, and metMb), and the physical light-scattering properties of the meat (Jeong et al., 2009; MacDougall, 1983). In addition, meat color is indirectly influenced by pH through its impact on the Mb chemical state (Fernández-López et al., 2004). In the present study, the meat color parameters of the samples from the three experimental groups were within the normal range, with relatively high b* values, indicating a slightly yellow color. This color characteristic is consistent with those reported by Van den Broeke et al. (2016).

Several studies have found that anti-GnRF vaccination does not result in meaningful alterations in meat color between intact and immunized gilts (Bohrer et al., 2014; Gamero-Negrón et al., 2015; Martinez-Macipe et al., 2016; Pérez-Ciria et al., 2021; Xue et al., 2019). No significant differences in L*, a*, b*, C*, or *h* were observed between the MIG samples and meat from the immunized gilts. Furthermore, varying the vaccination schedule did not significantly influence the meat color, suggesting that immunocastration and its timing do not affect pork color characteristics.

#### 4.2.3 Water holding capacity

The WHC of meat products is a vital attribute that affects product yield and has economic implications. In addition, it has an essential role in determining the eating quality of meat (Cheng & Sun, 2008). The WHC reflects the ability of meat to retain water under external forces (Perry, 2009). Most of the water is held in myofibrils and between muscle fibers, with a smaller fraction bound to proteins (Bond et al., 2004; Hamm, 1986). During postmortem, actomyosin cross-bridges reduce the intramyofibrillar space for water retention. In addition, the pH decrease reduces the repulsive forces between the protein molecules, decreasing the available space for water retention (Bendall & Swatland, 1988; Hamm, 1986; Kudryashov & Kudryashova, 2023). In this study, the WHC indicators—including drip loss, thawing loss, and cooking loss—were comparable across the three experimental groups, with no statistically meaningful variations observed. These results indicate that neither the administration of the anti-GnRF vaccine nor the vaccination schedule had a measurable impact on the protein-related water retention properties of the meat. These findings are consistent with previous studies that have reported no significant differences in WHC between intact and immunized gilts (Bohrer et al., 2014; Van den Broeke et al., 2016). Furthermore, WHC consistency among the treatment groups aligned with the pH data, which also showed no significant variation. As pH is a key factor influencing protein denaturation and water retention, the stable WHC supports the conclusion that muscle protein functionality is unaffected by immunocastration or vaccination timing.

#### 4.2.4 Meat tenderness

Warner–Bratzler shear force was used to measure meat tenderness, a key factor influencing consumer satisfaction, including mastication quality, repurchase decisions, and willingness to pay premium prices (Al Rharad et al., 2025; Choe et al., 2016; Lee et al., 2012; Warner et al., 2022). In this study, no significant tenderness differences were observed, indicating that neither the anti-GnRF vaccination nor the vaccination schedule affected meat tenderness. These findings are consistent with previous studies that have reported no influence of the anti-GnRF vaccine on this parameter (Bohrer et al., 2014; Martinez-Macipe et al., 2016). Furthermore, other studies have found that vaccination timing has no impact on meat tenderness between 7 and 12 weeks before slaughter (Pérez-Ciria et al., 2021). However, Van den Broeke et al. (2016) observed that the anti-GnRF vaccine decreases the shear force but has no effect on the sensory attributes of meat.

#### 4.2.5 Lipid oxidation (thiobarbituric acid reactive substance)

The TBARS method is widely used as an indicator of malonaldehyde, a primary intermediate lipid oxidation product (Wu et al., 2016). TBARS values did not differ markedly between the groups, indicating that anti-GnRF vaccination and its administration schedule have no discernible effects on lipid oxidation in pork. These findings are consistent with Gamero-Negrón et al. (2015), who reported no statistically significant differences in TBARS values between meat from immune-spayed and intact gilts. Albano-Gaglio et al. (2025) observed lower TBARS values in pork bellies with lower PUFA concentrations and higher fat deposition levels. This is consistent with the findings of Wood et al. (2008), who noted that higher PUFA concentrations increase the risk of lipid oxidation in pork. This suggests that TBARS is primarily influenced by PUFA content. In agreement with these previous studies, the present study showed no significant variations in TBARS values due to a lack of differences in PUFA levels among the samples from the three experimental groups—although there was a significant difference in the IMF content of the MIG samples and the meat from the immunized gilts.

### 4.3 Muscle histology

There have been few studies on the effects of anti-GnRF vaccination and the vaccination schedule on muscle histology in gilts. In the present study, all of the skeletal muscle components and associated connective tissue structures were observed for all three experimental groups, as shown in Fig. 2. The muscle histology plays a critical role in determining meat tenderness (Weston et al., 2002), with intramuscular connective tissue located in the perimysium and endomysium being a key contributing factor (Roy & Bruce, 2024). Several studies have reported strong positive correlations between perimysium thickness and shear force (Listrat et al., 2016). For example, Fang et al. (1999) observed a correlation coefficient of *r* = 0.98 in the semimembranosus muscle of pigs during growth, while Liu et al. (1996) reported a similar trend (*r* = 0.95) across six different types of chicken muscles. Additionally, research in beef muscles demonstrated that the biceps femoris, which has a larger perimysium area, exhibited higher shear force compared to the semimembranosus, which showed a smaller perimysium area (Dubost et al., 2013). These findings are consistent with the present study, in which no significant differences were observed in perimysium thickness or shear force values among the three experimental groups. Moreover, no significant differences in other histological parameters—including total fiber number, cross-sectional diameter, and cross section area—were found between the three groups, indicating that neither anti-GnRF vaccination nor variations in the vaccination schedule altered muscle microstructure. These findings align with the consistency observed in the meat quality parameters, including pH, color and WHC. Joo et al. (2013) emphasized the interdependence between the histological structure and meat quality traits and noted that various factors—including genetics, diet, environment, and hormones—influence muscle morphology. However, estrogen and progesterone were not identified as key regulators of muscle histology. This suggests that the immunocastration protocol used in the present study did not disrupt muscle development at the histological level.

## 5 Conclusion

This pilot study focused on meat characteristics, with the effects of anti-GnRF vaccination and administration age on nutritional composition, meat quality, and histological structure systematically evaluated. Under the experimental conditions, anti-GnRF vaccination administered to finishing gilts significantly increased the IMF content of their meat. Elevated IMF levels are closely associated with enhanced sensory attributes in meat and meat products, including taste, flavor, tenderness, and juiciness, making it a desirable and valuable attribute for the meat industry. However, the vaccination did not influence the proportions of the fatty acid profile of the IMF, the meat quality, or the histological structure of the muscle fibers. Furthermore, a comparison of vaccination schedules showed that early administration (at 9 and 13 weeks) produced no significant differences in the evaluated parameters compared with a typical vaccination schedule (at 12 and 16 weeks). These findings indicate that anti-GnRF vaccination enhances IMF content and that an earlier vaccination schedule may benefit farm management by enabling vaccination of smaller pigs and providing greater vaccination schedule flexibility.

However, this study is limited by a relatively small sample size, which may constrain the generalizability of the findings to the broader swine industry. To validate and strengthen these results, larger-scale studies are recommended, particularly if the ultimate goal is to inform potential modifications to commercial immunocastration protocols. Building upon the current findings, future research should also consider incorporating behavioral and physiological stress indicators to provide a more comprehensive assessment of the practical benefits of early immunocastration. Moreover, the assessment was limited to 24 weeks of age, aligning with the typical slaughter age in Thailand and Southeast Asia; thus, potential longer-term effects were beyond the scope of the present investigation.

## Data and model availability statement

None of the data were deposited in an official repository, but data can be provided by the corresponding author on request.

## CRediT authorship contribution statement

**Ditpon Kotatha:** Writing – review & editing, Writing – original draft, Visualization, Validation, Methodology, Investigation, Formal analysis. **Narut Thanantong:** Writing – review & editing, Validation, Formal analysis. **Sukanya Phuengjayaem:** Writing – review & editing, Validation, Formal analysis. **Bing-Zheng Li:** Writing – review & editing, Formal analysis. **Alongkot Boonsoongnern:** Supervision. **Autchara Kayan:** Supervision. **Montri Pattarapanawan:** Writing – review & editing, Writing – original draft, Visualization, Validation, Project administration, Methodology, Investigation, Funding acquisition, Formal analysis, Conceptualization.

## Declaration of competing interest

None.
